# Cardiac Magnetic Resonance-Verified Myocardial Fibrosis in Chagas Disease: Clinical Correlates and Risk Stratification

**DOI:** 10.5935/abc.20160168

**Published:** 2016-11

**Authors:** Marly Uellendahl, Maria Eduarda Menezes de Siqueira, Eveline Barros Calado, Roberto Kalil-Filho, Dário Sobral, Clébia Ribeiro, Wilson Oliveira, Silvia Martins, Jagat Narula, Carlos Eduardo Rochitte

**Affiliations:** 1 Setor de Ressonância Magnética Cardiovascular do Instituto do Coração (InCor) - Faculdade de Medicina da Universidade de São Paulo, São Paulo, SP; 2 Setor de Ressonância Magnética Cardíaca - Universidade Federal de São Paulo (UNIFESP/EPM), São Paulo, SP; 3 DASA/Delboni, São Paulo, SP; 4 Hospital Oswaldo Cruz - Universidade de Pernambuco - Departamento de Cardiologia, Recife, PE - Brazil; 5 Mount Sinai Hospital, Cardiology Department, Nova York - Estados Unidos

**Keywords:** Chagas Cardiomyopathy, Chagas Disease, Magnetic Resonance Spectroscopy, Risk factors, Prognosis

## Abstract

**Background:**

Chagas disease (CD) is an important cause of heart failure and mortality, mainly in Latin America. This study evaluated the morphological and functional characteristics of the heart as well the extent of myocardial fibrosis (MF) in patients with CD by cardiac magnetic resonance (CMR). The prognostic value of MF evaluated by myocardial-delayed enhancement (MDE) was compared with that via Rassi score.

**Methods:**

This study assessed 39 patients divided into 2 groups: 28 asymptomatic patients as indeterminate form group (IND); and symptomatic patients as Chagas Heart Disease (CHD) group. All patients underwent CMR using the techniques of cine-MRI and MDE, and the amount of MF was compared with the Rassi score.

**Results:**

Regarding the morphological and functional analysis, significant differences were observed between both groups (p < 0.001). Furthermore, there was a strong correlation between the extent of MF and the Rassi score (r = 0.76).

**Conclusions:**

CMR is an important technique for evaluating patients with CD, stressing morphological and functional differences in all clinical presentations. The strong correlation with the Rassi score and the extent of MF detected by CMR emphasizes its role in the prognostic stratification of patients with CD.

## Introduction

Although first described more than a hundred years ago,^1^ Chagas disease (CD) continues to be an important cause of morbidity and mortality in Latin America,^2,3^ affecting about 6 million people and with approximately 30 000 new cases detected every year.^4,5^ Due to constantly increasing migration of Latin Americans to North America and Western Europe, this disease is presenting a global challenge.^2^

The clinical outcomes in patients suffering from Chagas heart disease (CHD) are best expressed by risk factors including high functional New York Heart Association (NYHA) classes for symptomatic presentation, radiographic evidence of cardiomegaly, left ventricular (LV) dysfunction on echocardiography, non-sustained ventricular tachycardia (NSVT) on 24-hour ambulatory monitoring, low QRS voltage on electrocardiography, and male sex.^6-9^ Rassi et al^10^ have established a quantitative scoring system to identify CD patients at risk of dying prematurely. Six independent prognostic factors were identified, and each was assigned a number of points proportional to its regression coefficient: NYHA class III or IV (5 points), evidence of cardiomegaly on chest radiography (5 points), left ventricular systolic dysfunction on echocardiography (3 points), NSVT on 24-hour Holter monitoring (3 points), low QRS voltage on electrocardiography (2 points), and male sex (2 points). Risk scores were calculated for each patient and 3 risk groups were defined: low risk (0 to 6 points), intermediate risk (7 to 11 points), and high risk (12 to 20 points). The 10-year mortality rates for these 3 groups were 10%, 44%, and 84%, respectively, in a validation cohort.^10^ Chagas disease is pathologically characterized by post-inflammatory myocardial fibrosis (MF),^11-13^ which can be reliably identified by cardiac magnetic resonance (CMR) imaging using the myocardial-delayed enhancement technique (MDE).^14-16^ Since CMR-verified MF has been demonstrated to be an important predictor of arrhythmias and sudden death in several non ischemic cardiomyopathies, such as hypertrophic cardiomyopathy,^17^ and also in a pilot study of CD,^18^ we propose that a quantitative extent of MF should also offer prognostic value. The relationship between MF and the Rassi score,^10^ a validated prognostic score, in patients with and without apparent cardiomyopathy was evaluated in this study, what, by our knowledge, has not yet been performed systematically.

## Methods

Cardiac magnetic resonance was performed in 39 seropositive CD patients prospectively selected from the CD Clinic in Osvaldo Cruz Hospital from November 2004 to November 2006 (Table 1). Of these, 28 patients were asymptomatic and had normal ventricular function, being referred as indeterminate (IND) group; the remaining 11 symptomatic patients with systolic dysfunction were clinically deemed to have cardiomyopathy, the CHD group. All patients gave written informed consent for CMR imaging with MDE before inclusion, and the study was approved by the Institutional Research Review Committee. To calculate the Rassi score, all patients had a chest radiography, electrocardiogram, echocardiogram, 24-hour Holter and a clinical interview to check the NYHA functional class. The CMR was scheduled in a mean period of 30 days after those exams. Patients with previous myocardial infarction, clinical or laboratory evidence of ischemic heart disease, sustained ventricular tachycardia, heart valve disease, diabetes mellitus, hypercholesterolemia, pacemakers, implantable defibrillators and cerebrovascular clips were excluded.

**Table 1:** Patients’ characteristics

Characteristics	Group		All (n = 39)	p value
	IND (n = 11)	CHD (n = 28)		
Male sex	2 (18.2)	18 (64.3)	20 (51.3)	0.014*
Age (years)	48.3 ± 12.2	57.4 ± 12.5	54.8 ± 12.9	0.045†
Mean NYHA functional class	1 ± 0	2.2 ± 0.8	1.8 ± 0.9	< 0.001‡
NYHA functional class > 1	0 (0)	21 (75.0)	28 (71.8)	< 0.001*
LVEF (%)	57.9 ± 4.7	33.7 ± 16.5	40.5 ± 17.9	< 0.001 ‡
EDV (ml/m^2^)	64.5 ± 11.8	121.2 ± 62.2	105.2 ± 58.7	0.002 ‡
ESV (ml/m^2^)	26.7 ± 6.5	82.8 ± 55.5	67.0 ± 53.4	< 0.001 ‡
LV mass (g/m^2^)	46.7 ± 21.7	79.5 ± 34.6	70.2 ± 34.6	0.006 ‡
RVEF (%)	41.5 ± 13.8	35.2 ± 12.5	37.0 ± 13.0	NS

Data are expressed as mean ± SD or number (%).

* Fisher’s exact test;

† Student’s t test;

‡ Kruskal-Wallis test. IND: indeterminate; CHD: Chagas heart disease; NYHA: New York Heart Association; LVEF: left ventricular ejection fraction; EDV: end diastolic volume; ESV: end-systolic volume; RVEF: right ventricular ejection fraction; NS: not significant.

### Magnetic resonance imaging methods

Patients underwent MRI examination on 1.5-T Signa GE system (Wakeusha, Wisconsin). Short and long axis images of the heart were obtained during breath hold and with an electrocardiogram-triggered pulse sequences. The first sequence was a gradient-echo steady-state free precession (SSFP) to assess LV and right ventricular (RV) morphology and function. The second sequence was an inversion-recovery prepared segmented gradient-echo to obtain MDE,^19^ 10 to 20 minutes after an intravenous bolus of 0.2 mmol/kg of gadolinium-based contrast (Dotarem®, gadoteric acid - Gd-DOTA, Guerbet Aulnay-Sous-Bois - France). For the cine images, using SSFP sequence, the parameters were: repetition time 3.4ms, echo time 2.0ms, flip angle 45º, matrix 256x160, cardiac phases 20, views per segment 8 to 16 to obtain a temporal resolution of 55ms or less, slice thickness 8mm, gap between slices 2mm, and field of view 36 to 40 cm. For the MDE pulse sequence, the following parameters were used for short and long axis: repetition time 7.3 ms, echo time 3.2 ms, flip angle 25º, matrix 256 x 196, slice thickness 8 mm, gap between slices 2 mm and field of view 36 to 40 cm, inversion time 200 ms to 300 ms, receiver bandwidth 32.5 kHz, every RR acquisition and number of excitations 2. Short-axis views were prescribed from base to apex (usually 8 to 12 cine slices/heart) perpendicular to the ventricular long-axis covering the entire left ventricle. Importantly, all slice locations were exactly the same for both pulse sequences, allowing comparison of function and morphology with the tissue characterization provided by MDE. Additional slices were acquired as needed to provide complete coverage of very large ventricles.

### Data analyses

The endocardial and epicardial borders of the myocardium were planimetered on the short-axis cine images. All measurements were done manually. The true 3D volumetric data was obtained by the summation of the areas of short axis, without any geometric assumption, known as Simpson's method. The end-systolic and diastolic frames were identified by determining the ventricular blood-pool areas. Left ventricular volumes were derived by summation of blood-pool areas, and the ejection fraction (EF) was calculated accordingly. All CMR analyses were performed with Siemens Argus Software (Siemens AG, Munich, Germany). On the MDE short-axis images, the segmental transmurality extent of MDE, as percentage of LV mass, was measured using the semi-quantitative method described previously by Azevedo Filho et al.,^20^ scored by 2 observers as the visual percent area of the enhanced segment. The semi-quantitative method consisted of the visual evaluation of all short-axes slices and condense the data into 8 short-axes representative of the left ventricle divided into 48 segments as follows: slice 1 and 2 (representing all apical slices) with 4 segments each; slices 3 to 6 (middle slices) with 6 segments each; and slices 7 and 8 (basal slices) with 8 segments each. All segments received a score according to the percentage of involvement obtained on MDE (0, 1, 2 or 3). Score 0 corresponded to absence of MDE, score 1 corresponded to 1% to 25% of MDE, score 2 corresponded to 26% to 75% of MDE, and score 3 corresponded to greater than 75% of MDE of the area of the segment involved. Additionally, we identified different MDE patterns as apical isolated, multifocal and diffuse.

### Statistical analyses

Normally distributed continuous variables were compared by the unpaired Student *t* test and one-way analysis of variance with the Bonferroni test for multiple comparisons. The Fisher exact test was used for proportions comparisons. The non-parametric test for discrete variables and non-normal continuous variables was Kruskal-Wallis rank test. Normality was determined by Shapiro-Francia W' test. Simple linear regression was used between the MF mass and LVEF; MF and Rassi score; and LVEF and Rassi score. Given the exploratory nature of the study, formal calculations of sample size were not performed. Based on previous articles of our group, which investigated MF in patients with similar characteristics, we chose to include more than 30 patients. Stata 8.0 (Stata Corp., College Station, Texas) was used, and p < 0.05 (two-tailed) was considered statistically significant.

## Results

**Figure 1:**
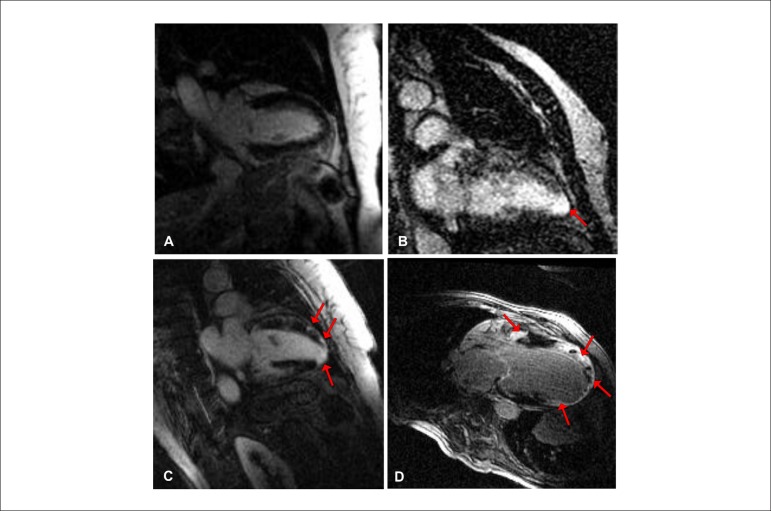
Location and severity of myocardial fibrosis (MF) in Chagas disease. A) no MF; B) isolated apical MF; C) multifocal MF; and D) diffuse MF.

Patients in the CHD group were older and showed higher frequency of male sex than the IND group (Table 1). CHD patients were at least NYHA functional class II symptomatic, and their CMR imaging demonstrated moderate-to-severe global LV dysfunction. IND subjects showed either normal LV function or mild LV dysfunction. The majority of imaging variables showed substantial differences between the 2 groups, even when 9 patients with NSVT were excluded from analysis. Only RVEF did not show significant variation between groups. Qualitative MF was detected by CMR in 29 patients (72%); greater proportion of CHD patients (89%) compared to IND subjects (27%) demonstrated MDE evidence of MF (p <0.001). All 9 patients with NSVT had MF detected by CMR imaging. Different patterns, location and severity of MF were detected as shown in Figure 1.

**Figure 2:**
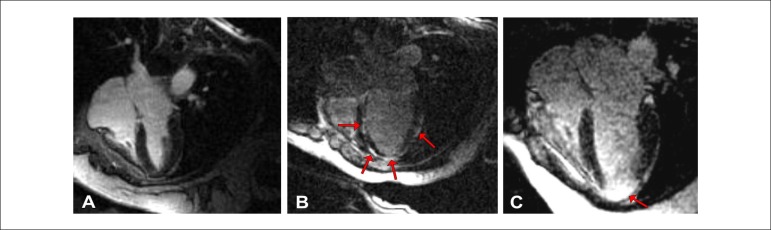
Patterns of myocardial fibrosis: A) no myocardial fibrosis detected (IND group); B) multifocal and subepicardial myocardial fibrosis (CHD group); C) severe myocardial fibrosis at the apex (patient with non-sustained ventricular tachycardia).

The presence of MF demonstrated a direct relationship with the clinical severity of the disease when we compared the 2 groups (Table 2). Significant differences were found among the NYHA functional class: NYHA II/III (100%) for CHD group vs. NYHA I (39%) for IND group (p = 0.001, Fisher's exact test), and also there were more male patients with MF (96%) vs. female patients (47%) (p = 0.001) (Table 3). More importantly, we found that the quantitative assessment of MF, described as a percentage of LV mass, was statistically higher in the CHD group (25.3±1.4%) compared to IND group (0.63 ± 1.4%) (Table 4 and Figure 2). Furthermore, the percentage of MF was substantially higher in CHD group in patients with arrhythmic presentation (30.6 ± 18.6%) as compared to those who did not have significant ventricular arrhythmias (22.7 ± 19.7%).

**Table 2:** Myocardial fibrosis detected by magnetic resonance imaging and its relation in IND and CHD groups

	Groups		All(n = 39)	p value
	IND(n = 11)	CHD(n = 28)		
No MF detected	8 (72.7)	3 (10.7)	11 (28.2)	< 0.001*
MF detected	3 (27.3)	26 (89.3)	29 (71.8)	

Data are expressed as number (%).

* Fisher’s exact test. MF: myocardial fibrosis; IND: indeterminate; CHD: Chagas heart disease.

**Table 3:** Detection of myocardial fibrosis in relation to patients’ characteristics, clinical data and morphologic and functional assessment by magnetic resonance imaging

Characteristics	Group		All (n = 39)	p value
	No fibrosis (n = 11)	With fibrosis (n = 28)		
Male sex	1 (9.1)	19 (67.9)	20 (51.3)	0.001*
Age (yrs)	56.1 ± 11.8	54.4 ± 13.5	54.8 ± 12.9	NS†
LVEF (%)	60.2 ± 4.9	32.8 ± 14.9	40.5 ± 17.9	< 0.001‡
EDV (ml/m^2^)	61.6 ± 13.1	122.3 ± 61.0	105.2 ± 58.7	< 0.001‡
ESV (ml/m^2^)	23.8 ± 6.0	83.9 ± 54.3	67.0 ± 53.4	< 0.001‡
RVEF (%)	40.3 ± 13.5	35.8 ± 12.8	37.0 ± 13.0	NS‡
NYHA functional class > 1	0 (0)	21 (75)	21 (53.8)	< 0.001*
NYHA functional class = 1	11 (100)	7 (25)	18 (46.1)	< 0.001*

Data are expressed as mean ± SD or number (%).

* Fisher’s exact test;

† Student’s t test;

‡ unpaired Kruskal-Wallis test. LVEF: left ventricular ejection fraction; EDV: end-diastolic volume; ESV: end-systolic volume; RVEF: right ventricular ejection fraction; NYHA: New York Heart Association.

**Table 4:** Percentage of myocardial fibrosis in clinical forms

	IND (n = 11)	CHD		All	p value
		NSVT not detected (n = 19)	NSVT detected (n = 9)		
Myocardial fibrosis (%)	0.63 ± 1.4	22.7 ± 19.7	30.6 ± 18.6	18.3 ± 19.8	< 0.001*

Data are expressed as mean ± SD;

* ANOVA. NSVT: non-sustained ventricular tachycardia; IND: indeterminate; CHD: Chagas heart disease.

**Figure 3:**
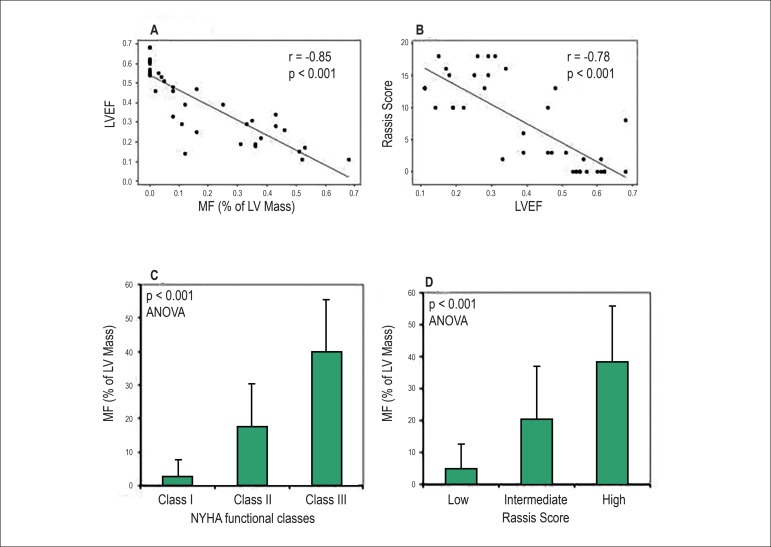
A) Inverse correlation of left ventricular ejection fraction (LVEF) and myocardial fibrosis (MF); B) Correlation between Rassi scores and LVEF; C) Mean MF increases in relation to New York Heart Association (NYHA) functional classes (p < 0.001 by analysis of variance); D) Left ventricular (LV) fibrotic mass and Rassi scores.

Quantitative estimates of MF correlated significantly with LV function and NYHA functional class (Figures 3A and 3C). Myocardial fibrosis had an inverse correlation with myocardial function: patients with the highest percentage of fibrotic mass had the lowest LVEF (Figure 3A; r = -0.85 and, p< 0.001). On top of that, patients with fibrotic mass greater than 10% of the ventricular mass demonstrated significantly greater impairment of LVEF as compared with the patients with lesser fibrotic mass (26.6 ± 12.3% vs. 55.3 ± 8.3%, respectively, p < 0.001).

CMR-based LVEF showed an inverse correlation with Rassi scores (r= -0.78, p < 0.001) (Figure 3B). Myocardial fibrosis also showed a strong positive correlation with the Rassi score (r= 0.76, p < 0.001; Figure 3D). This correlation was reinforced by subgroup analysis showing progressive MF from low to high-risk Rassi score groups. The percentages of MDE-related MF in low, intermediate and high Rassi risk groups were 5.0±7.7%, 19.8±17.3%, and 38.1±17.7%, respectively (p<0.001, Figure 3D). The MF location also correlated significantly with Rassi scores (Table 5). Different patterns of MF, including no fibrosis, isolated MF at the apex, multifocal or diffuse fibrosis are illustrated in Figure 1.

**Table 5:** Average Rassi score and location of myocardial fibrosis (MF)

	No MF detected (n = 12)	Apical MF (n = 10)	Multifocal MF (n = 5)	Diffuse MF (n = 12)	All (n = 39)	p value
Average Rassi score	1 ± 2.3	6.2 ± 6.1	8.6 ± 6.4	14.1 ± 3.1	7.3 ± 6.7	< 0.001*

Data are expressed as mean ± SD;

* Kruskal-Wallis test.

## Discussion

This study establishes the relationship between the clinical severity and a clinical prognostic score (Rassi score) with CMR-based MF in CD. Myocardial fibrosis was detected in 25 out of 28 patients (89%) with clinical cardiomyopathy (CHD group), reflecting its role in the severity of CD. Myocardial fibrosis was found to be greater in male patients compared with female, and may underlie higher reported mortality in men.^10^ Myocardial fibrosis correlated strongly with the NYHA functional class and LV function. A maximum MF was identified in a subgroup of patients presenting with NSVT (30.0±18.6%). This finding may strengthen the hypothesis that MF in CD is related to arrhythmias and sudden death, which has also been suggested by previous work relating MF and electrophysiological study.^18^ On the other hand, patients with lack of clinical cardiomyopathy (IND group) had significantly less extensive MF (0.63±1.4%), confirming two previous MRI studies^15,16^ and one pathological study^21^ that had shown only minor and focal inflammation and fibrosis in the so-called indeterminate CD presentation. The percentage of MF based on MDE-CMR was progressively and strongly associated with the clinical severity or Rassi score (r = 0.76).

In this study, all patients without apparent cardiomyopathy (IND group) had an echocardiogram with normal RV function. Cardiac magnetic resonance is considered more sensitive and accurate than echocardiography for RVEF measurements. Cardiac magnetic resonance showed that some of these patients had RV dysfunction (RVEF% = 41.5 ± 13.8), but it is important to point out that, regarding CMR measurements, in several references in literature and in our clinical routine the normal range for RVEF frequently extends to around 45%.^22^ Therefore, our values might represent low normal values for RVEF or mild RV dysfunction. The reason for that in the IND group is unknown. However, impairment of RV function is more common in chronic CD than in other forms of heart failure. We have not analyzed the MDE in the right ventricle due to the difficulty to evaluate the thin RV wall. Right ventricular MF, as well as other mechanisms, such as autonomic dysfunction may be involved in RV dysfunction in CD.

From the viewpoint of clinical investigation, we believe that CMR can evaluate in a more refined way those patients with mild global or segmental myocardial dysfunction that could not be detected in the routine evaluation by echocardiography, especially in relation to RV function, and this may help future research investigating heart failure in CD. In addition, patients with ventricular arrhythmias and fibrosis detected by MDE on CMR, similarly to other cardiomyopathies, could be evaluated as potential candidates for antiarrhythmic therapy. We also believe that MRI techniques under development, such as T1-Mapping may be useful in the detection of interstitial fibrosis and the investigation of drugs that can prevent the progression of cardiac dysfunction, myocardial inflammation and fibrosis.

This current clinical investigation is not a longitudinal study and the sample size is relatively small, but within the range of most prior CMR studies in CD. This study was specially challenging, because investigated patients of low socioeconomic level, mostly living in rural areas, and with difficulties to access our MRI scanner. Nonetheless, MRI studies even with smaller sample sizes, such as this, can achieve statistical significance due to the MRI lower variability measurements. Therefore, this study found a strong and significant correlation (r = 0.8) between Rassi score and MF mass despite its relatively small sample size.

To our knowledge, this is the first study involving the quantification of MF by CMR and prognostic data, in this case obtained by evaluating the Rassi score (which uses several validated markers of worse prognosis, such as male sex, low voltage on electrocardiogram, enlarged heart silhouette on chest x-ray, heart dysfunction by echocardiography and the presence of NSVT on Holter monitoring). As previously demonstrated by Rochitte et al.^15^ in CD patients from southeast Brazil, our data confirmed a strong correlation between MF and dysfunction. However, in the study by Rochitte et al., prognostic data, such as Rassi score used as a prognostic maker, were not available. Our current manuscript presents original data demonstrating a strong correlation between the quantification of MF mass by CMR and Rassi score, a prognostic indicator validated in different CD populations. This suggests that CMR may be a powerful tool in the evaluation of chagasic patients, to identify those patients at higher risk of cardiovascular events and may help the prognostic stratification for implantable cardioverter-defibrillator candidates. Our study data support future larger and longitudinal studies to investigate the prognostic value of CMR in CD.

## Conclusions

CMR-verified MF is significantly increased in CD patients with clinical cardiomyopathy. Myocardial fibrosis quantification shows a strong relationship with Rassi score, a well-validated prognostic score for CD. Cardiac magnetic resonance-verified MF deserves to be investigated as an independent prognostic factor, emphasizing its value as a prognostic tool for the risk stratification in this disease.
